# Predictive value of serum magnesium levels for prognosis in patients with non-small cell lung cancer undergoing EGFR-TKI therapy

**DOI:** 10.1515/biol-2022-0923

**Published:** 2024-07-24

**Authors:** Fang-Zhou Xu, Fu-Rong Meng, Wan-Jing Li, Lu Xu, Hao Zhang, Yan-Bei Zhang, Xiao-Yun Fan

**Affiliations:** Department of Geriatric Respiratory and Critical Care Medicine, The First Affiliated Hospital of Anhui Medical University, Hefei, 230032, Anhui, China; Anhui Geriatric Institute, Hefei, 230001, Anhui, China; Department of Geriatric Respiratory and Critical Care Medicine, The First Affiliated Hospital of Anhui Medical University, No. 218 of JiXi Road, ShuShan District, Hefei, 230032, Anhui, China

**Keywords:** EGFR-TKI, magnesium, non-small cell lung cancer, overall survival, progression-free survival

## Abstract

The aim of this study is to assess the impact of serum magnesium (Mg) levels on prognostic outcomes in patients with non-small cell lung cancer (NSCLC) undergoing treatment with epidermal growth factor receptor tyrosine kinase inhibitors (EGFR-TKI). A cohort comprising 91 patients with NSCLC with epidermal growth factor receptor mutations received EGFR-TKI therapy. Assessments of liver and kidney function and electrolyte levels were conducted before treatment initiation and after completing two cycles of EGFR-TKI therapy. Data on variables such as age, gender, presence of distant metastasis, smoking history, other therapeutic interventions, and the specific TKI used were collected for analysis. Cox regression analysis revealed that patients with higher Mg levels prior to EGFR-TKI therapy had significantly longer progression-free survival (PFS) and overall survival (OS). Elevated Mg levels remained predictive of PFS and OS after two cycles of EGFR-TKI therapy. Multiple regression analysis confirmed these findings. Additionally, it was observed that smokers might represent a unique population, demonstrating a correlation between OS and Mg levels. Our findings indicate that serum Mg level is a prognostic factor in patients with NSCLC undergoing EGFR-TKI therapy. This may provide new insights into the underlying mechanisms of EGFR-TKI therapy related to electrolyte balance.

## 1 Introduction

Non-small-cell lung cancer (NSCLC) originates in the epithelial cells lining the pulmonary airways and includes distinct histological subtypes: adenocarcinoma, squamous cell carcinoma, and large cell carcinoma. NSCLC is the most prevalent form of lung cancer, significantly contributing to cancer-associated morbidity and mortality. The introduction of targeted therapeutic interventions and lifestyle modifications has reduced the impact of lung cancer on human health [1]. Targeted and immunotherapy-based treatments have notably improved survival rates for patients with NSCLC [2]. Since 1991, reports from the American Cancer Society and Cancer Statistics Center have shown a steady decline in the cancer mortality rate, with lung cancer experiencing the most pronounced decline among the four leading cancers between 2014 and 2018 [3].

Despite the effectiveness of targeted therapy for NSCLC, drug resistance remains a significant challenge. Throughout the treatment course with first- and second-generation epidermal growth factor receptor tyrosine kinase inhibitors (EGFR-TKI), a substantial proportion of patients experience disease progression due to inadequate therapeutic responses against the T790M mutation and other acquired resistance mechanisms [4]. The expanding array of targeted pharmacotherapeutic agents approved for clinical use has significantly enhanced the management of patients with NSCLC with metastatic EGFR mutations, particularly those harboring the acquired EGFR T790M mutation, who show progression despite ongoing EGFR-TKI treatment [4,5]. Early detection of disease progression and information about drug resistance are crucial for timely drug adjustments and essential for prolonging patient survival and enhancing the quality of life.

The strong association between magnesium (Mg) levels and the severity of various chronic diseases, some of which occur as comorbidities, underscores the importance of Mg levels as a critical observational marker. Reduced serum Mg concentrations have been linked to elevated risks of prediabetes and diabetes [6]. Lötscher et al. demonstrated that Mg enhances T cell immunity and that low serum Mg concentrations are associated with poorer outcomes in cancer immunotherapy, including chimeric antigen receptor T cell therapy [7]. Frequent hypomagnesemia is a significant predictor of decreased survival in patients with head and neck cancer undergoing chemoradiotherapy [8]. However, the relationship between hypomagnesemia and the prognosis of EGFR-TKI therapy for NSCLC has been infrequently studied. We hypothesize that Mg levels correlate with prognosis following EGFR-TKI therapy.

## 2 Method

### 2.1 Patient enrollment

From March 2016 to October 2021, 91 patients with NSCLC and EGFR mutations received EGFR-TKI therapy at the First Affiliated Hospital of Anhui Medical University. The inclusion criteria for this study were as follows: (i) a confirmed diagnosis of NSCLC with a clear pathological type; (ii) next-generation sequencing to identify specific genetic mutations; and (iii) completion of more than two cycles of oral EGFR-TKI therapy, with the first effective evaluation conducted after 60 days of treatment. The exclusion criteria were as follows: (i) patients with histologically mixed small cell carcinoma or small cell carcinoma; (ii) patients who were lost to follow-up or deceased within two months of treatment initiation; and (iii) patients with an active infection or kidney disease, as these conditions could affect hematology laboratory marker values.


**Informed consent:** Informed consent has been obtained from all individuals included in this study.
**Ethical approval:** The research related to human use has been complied with all the relevant national regulations, institutional policies and in accordance with the tenets of the Helsinki Declaration, and has been approved by the Ethics Committee of The First Affiliated Hospital of Anhui Medical University (No.Quick-PJ 2023-12-60).

### 2.2 Data statistics

Overall survival (OS) was the primary endpoint, while progression-free survival (PFS) was the secondary endpoint. OS was measured from the initiation of EGFR-TKI therapy until all-cause mortality or the date of the last follow-up. PFS was defined as the period from the initiation of TKI therapy to disease progression, tumor metastasis, or all-cause death. Evaluations were conducted monthly during the initial two months following the commencement of EGFR TKI therapy, and subsequently, at three-month intervals. Comprehensive assessments were promptly conducted upon the exacerbation or emergence of new symptoms. The amalgamation of clinical data with findings from chest computed tomography (CT) scans was utilized to appraise therapeutic efficacy, in accordance with the Response Evaluation Criteria in Solid Tumors (RECIST, version 1.1).

Variables extracted included age, gender, distant metastasis, smoking history, body mass index (BMI), other treatments (thoracic surgery, chemotherapy, radiofrequency ablation, and radiotherapy), concomitant diseases (stroke, arterial hypertension, diabetes, and cardiopathy), and the choice of TKI. Non-smokers were defined as patients who had never smoked, while smokers were defined as those who had a history of smoking or had not quit smoking at the time of their cancer diagnosis. Additionally, liver and kidney function, as well as electrolyte levels (including Mg, calcium (Ca), phosphorus (P), potassium (K), blood urea nitrogen (BUN), serum creatinine (SCr), alanine aminotransferase (ALT), and aspartate aminotransferase (AST)), was evaluated prior to and after two cycles of TKI treatment.

### 2.3 Statistical analyses

Continuous variables are expressed as means ± standard deviations. Univariate prognostic variables were screened using Cox regression analysis. The relationship between Mg levels and PFS and OS before and after therapy was evaluated using a multiple logistic regression model with adjustments for confounding factors. Hazard ratios (HR) are reported as relative risks with corresponding 95% confidence intervals (CI) and were derived using Cox regression analysis. Survival curves were plotted using the Kaplan–Meier method and log-rank test. The optimal cutoff Mg level was determined using the surv_cutpoint algorithm of the survival R package [9,10]. Finally, interactions between the Mg level and the other variables were examined. Statistical significance was set at *P* < 0.05. Statistical analyses were conducted using Empower^®^ (www.empowerstats.com; X&Y Solutions, Inc., Boston, MA, USA), R (http://www.R-project.org), and R software (version 4.2.0) [11].

## 3 Results

### 3.1 Patient demographics

This study included 91 NSCLC patients who underwent EGFR-TKI therapy. Comprehensive patient demographic data are presented in Table 1. The median age of the patients was 61.57 years, and 52.75% were male. Among the cohort, 44 patients (48.35%) received chemotherapy prior to oral TKI treatment. Additionally, six patients (6.59%) underwent radiofrequency ablation, two patients (2.20%) underwent thoracotomy, and three patients (3.30%) underwent radiotherapy. Gefitinib was the most frequently prescribed EGFR-TKI, followed by icotinib and erlotinib. Thirty-one patients were diagnosed with arterial hypertension, 22 with diabetes, seven had a history of stroke, and four had a history of coronary heart disease.

**Table 1:** Demographics characteristics of 91 patients with NSCLC

Characteristics	Mean ± SD	Characteristics	Mean ± SD
Age	61.57 ± 10.64	**Coronary heart disease**	
ALT(U/L)	38.19 ± 156.69	No	87 (95.60%)
AST(U/L)	28.21 ± 75.12	Yes	4 (4.40%)
BUN (mmol/L)	5.27 ± 2.34	**Stroke**	
SCr (μmol/L)	66.65 ± 14.69	No	84 (92.31%)
K (mmol/L)	4.00 ± 0.35	Yes	7 (7.69%)
Ca (mmol/L)	2.26 ± 0.13	**Chemotherapy**	
P (mmol/L)	1.12 ± 0.19	No	47 (51.65%)
Mg (mmol/L)	0.88 ± 0.07	Yes	44 (48.35%)
BMI	22.4 ± 3.4	**Radiofrequency ablation**	
**Gender**		No	85 (93.41%)
Female	43 (47.25%)	Yes	6 (6.59%)
Male	48 (52.75%)	**Radiotherapy**	
**Metastasis**		No	88 (96.70%)
No	7 (7.69%)	Yes	3 (3.30%)
Yes	84 (92.31%)	**Operation**	
**Smoke**		No	89 (97.80%)
No	66 (72.53%)	Yes	2 (2.20%)
Yes	25 (27.47%)	**Drug**	
**Hypertension**		Gefitinib	42 (46.15%)
No	60 (65.93%)	Anlotinib	3 (3.30%)
Yes	31 (34.07%)	Icotinib	27 (29.67%)
**Diabetes**		Erlotinib	9 (9.89%)
No	69 (75.82%)	Osimertinib	5 (5.49%)
Yes	22 (24.18%)	Afatinib	4 (4.40%)
		Dacomitinib	1 (1.10%)

ALT, glutamic pyruvic transaminase; AST, glutamic oxaloacetic transaminase; BUN, blood urea nitrogen; SCr, serum creatinine; Ca, calcium; Mg, magnesium; P, phosphorus; K, potassium; BMI, body mass index.

### 3.2 Prognostic factors before EGFR-TKI therapy

The results of the univariate analysis of liver function, renal function, electrolytes, and medical history prior to therapy are displayed in Table 2. Cox regression analysis revealed that age and gender had little influence on OS and PFS. Renal function and other common electrolytes, such as K, P, and Ca, also had negligible effects on prognosis. ALT and AST had a negligible impact on PFS but had a protective effect on OS. Hypertension, diabetes, history of cardiovascular and cerebrovascular diseases, and history of smoking were risk factors for prognosis after EGFR-TKI therapy, though this trend was not statistically significant. Patients with higher Mg levels before EGFR-TKI therapy had significantly higher PFS and OS (*P* = 0.0100 and *P* = 0.0092, respectively). Multiple regression analysis using the Cox survival model confirmed this result (Table 3). After adjusting for variables such as gender, age, SCr, BUN, ALT, AST, disease history, and other treatments, the predictive effect on PFS and OS remained significant (HR = 0.02; 95% CI = 0.00–0.05; P = 0.0216; HR = 0.00; 95% CI = 0.00–0.06; *P* = 0.0017).

**Table 2:** Prognostic factors before EGFR-TKI treatment

	Statistics	PFS HR (95% CI) *P* value	OS HR (95% CI) *P* value
**Gender**			
Female	43 (47.25%)	1	1
Male	48 (52.75%)	0.85 (0.54, 1.32) 0.4617	0.64 (0.35, 1.18) 0.1519
Age	61.57 ± 10.64	0.99 (0.97, 1.01) 0.2870	1.02 (1.00, 1.05) 0.1032
**Metastasis**			
No	7 (7.69%)	1	1
Yes	84 (92.31%)	1.50 (0.61, 3.74) 0.3784	1.06 (0.33, 3.45) 0.9177
ALT(U/L)	28.21 ± 75.12	1.00 (1.00, 1.00) 0.5770	0.96 (0.92, 1.00) 0.0829
AST(U/L)	38.19 ± 156.69	1.00 (1.00, 1.00) 0.6007	0.98 (0.95, 1.00) 0.0779
BUN (mmol/L)	5.27 ± 2.34	1.03 (0.93, 1.15) 0.5183	1.06 (0.92, 1.22) 0.4023
SCr (μmol/L)	66.65 ± 14.69	1.00 (0.98, 1.02) 0.8521	1.01 (0.99, 1.03) 0.4963
K (mmol/L)	4.00 ± 0.35	1.09 (0.58, 2.06) 0.7841	1.14 (0.45, 2.87) 0.7774
Ca (mmol/L)	2.26 ± 0.13	0.56 (0.09, 3.33) 0.5232	0.39 (0.04, 3.80) 0.4177
P (mmol/L)	1.12 ± 0.19	0.83 (0.25, 2.75) 0.7608	0.77 (0.16, 3.79) 0.7520
Mg (mmol/L)	0.88 ± 0.07	0.02 (0.00, 0.37) 0.0100	0.00 (0.00, 0.26) 0.0092
BMI	22.4 ± 3.4	1.04 (0.97, 1.11) 0.2355	1.03 (0.94, 1.13) 0.5683
**Smoke**			
No	66 (72.53%)	1	1
Yes	25 (27.47%)	1.20 (0.73, 1.97) 0.4786	1.44 (0.75, 2.77) 0.2751
**Diseases history**			
No	55 (60.44%)	1	1
Yes	36 (39.56%)	1.11 (0.70, 1.74) 0.6645	1.10 (0.60, 2.04) 0.7564
**Other treatment**			
No	43 (47.25%)	1	1
Yes	48 (52.75%)	1.05 (0.66, 1.65) 0.8504	0.63 (0.34, 1.18) 0.1474

**Table 3:** Multiple regression equation of magnesium before EGFR-TKI treatment

	Adjust I	Adjust II
PFS	0.02 (0.00, 0.51) 0.0179	0.02 (0.00, 0.55) 0.0216
OS	0.00 (0.00, 0.08) 0.0023	0.00 (0.00, 0.06) 0.0017

Adjust I model – adjust for gender and age.

Adjust II model – adjust for gender, age, SCR, BUN, ALT, AST, diseases history, and other treatment.

### 3.3 Survival curves before EGFR-TKI therapy

**Figure 1:**
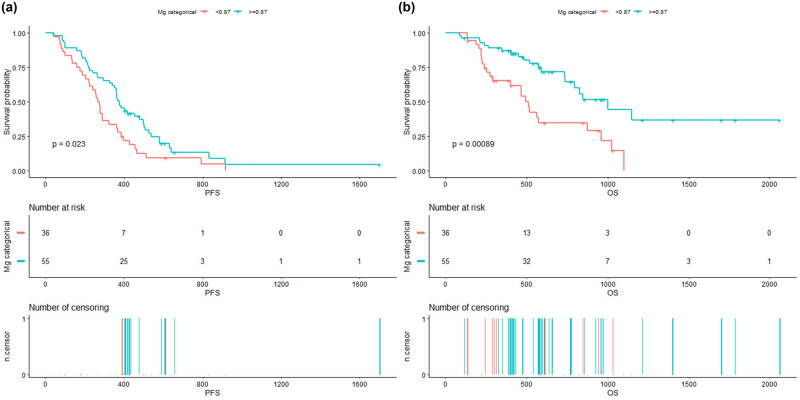
Survival curves before EGFR-TKI therapy according to Mg level: (a) PFS before EGFR-TKI treatment; (b) OS before EGFR-TKI treatment.

Using the surv_cutpoint function in the survival R package, we determined the optimal cutoff Mg level for OS, which was 0.87 mmol/L based on the 91 patients in the study cohort. Consequently, the patients were divided into the high and low Mg level groups, comprising 55 (60.44%) and 36 (39.56%) patients, respectively. The median PFS in the high Mg group was 369 days (range: 323–478 days) compared to that of 275 days (range: 223–363 days) in the low Mg group. The survival curve analysis showed that PFS was significantly higher in the high Mg group than in the low Mg group (*P* = 0.023; Figure 1). Similarly, OS was higher in the high Mg group (mean: 1,026 days; range: 825–NA days) compared to that in the low Mg group (mean: 517 days; range: 450–846 days). Moreover, the difference in OS between the high Mg and low Mg groups was highly statistically significant (*P* = 0.00089).

### 3.4 Prognostic factors after two EGFR-TKI treatment cycles

Given the excellent pre-treatment data, we further evaluated changes in liver and kidney function, electrolytes, and other parameters after two treatment cycles (Table 4). A univariate analysis of liver function, renal function, and electrolytes post-treatment revealed that most data indicating a decreased risk of disease progression was related to electrolyte levels, particularly Ca and Mg. Notably, only Mg remained statistically significant in terms of PFS, with an HR of 0.01 (95% CI: 0.00–0.07; *P* = 0.0001), whereas the *P* value associated with calcium was greater than 0.05, indicating a lack of statistical significance.

**Table 4:** Prognostic factors after two cycles of EGFR-TKI treatment

	Statistics	PFS HR (95% CI) *P* value	OS HR (95% CI) *P* value
**Gender**			
Female	43 (47.25%)	1	1
Man	48 (52.75%)	0.85 (0.54, 1.32) 0.4617	0.64 (0.35, 1.18) 0.1519
Age	61.57 ± 10.64	0.99 (0.97, 1.01) 0.2870	1.02 (1.00, 1.05) 0.1032
**Metastasis**			
No	7 (7.69%)	1	1
Yes	84 (92.31%)	1.50 (0.61, 3.74) 0.3784	1.06 (0.33, 3.45) 0.9177
ALT(U/L)	30.04 ± 20.20	1.00 (0.99, 1.01) 0.5812	1.00 (0.98, 1.01) 0.5103
AST(U/L)	27.27 ± 14.01	1.01 (1.00, 1.03) 0.1704	1.01 (0.98, 1.03) 0.6172
BUN (mmol/L)	5.37 ± 1.82	1.00 (0.88, 1.14) 0.9470	1.07 (0.89, 1.27) 0.4864
SCr (μmol/L)	71.18 ± 19.67	1.00 (0.98, 1.01) 0.7131	1.00 (0.98, 1.02) 0.9446
K (mmol/L)	4.05 ± 0.67	0.94 (0.68, 1.30) 0.7101	0.88 (0.51, 1.52) 0.6437
Ca (mmol/L)	2.22 ± 0.13	0.17 (0.03, 1.09) 0.0618	0.01 (0.00, 0.22) 0.0023
P (mmol/L)	1.12 ± 0.20	1.31 (0.42, 4.13) 0.6428	0.79 (0.17, 3.70) 0.7696
Mg (mmol/L)	0.86 ± 0.09	0.01 (0.00, 0.07) 0.0001	0.01 (0.00, 0.42) 0.0154
BMI	22.4 ± 3.4	1.02 (0.96, 1.09) 0.4576	1.00 (0.98, 1.01) 0.5103
**Smoke**			
No	66 (72.53%)	1	1
Yes	25 (27.47%)	1.20 (0.73, 1.97) 0.4786	1.44 (0.75, 2.77) 0.2751
**Diseases history**			
No	55 (60.44%)	1	1
Yes	36 (39.56%)	1.11 (0.70, 1.74) 0.6645	1.10 (0.60, 2.04) 0.7564
**Other treatment**			
No	43 (47.25%)	1	1
Yes	48 (52.75%)	1.05 (0.66, 1.65) 0.8504	0.63 (0.34, 1.18) 0.1474

For OS, changes in electrolyte levels were the most significant, with Mg and Ca being statistically significant factors. High Ca levels were protective for prognosis (HR = 0.01; 95% CI: 0.00–0.22; *P* = 0.0023). Since Ca values were only significant for OS after therapy, no additional research was conducted on this parameter. Multiple regression analysis using the Cox survival model (Table 5) adjusted for variables such as gender, age, SCr, BUN, ALT, AST, disease history, and other treatments. Changes in Mg levels continued to exhibit a predictive effect on both PFS (HR = 0.00; 95% CI: 0.00–0.06; *P* < 0.0001) and OS (HR = 0.00; 95% CI: 0.00–0.20; *P* = 0.0056).

**Table 5:** Multiple regression equation of Mg after two cycles of EGFR-TKI treatment

	Adjust I	Adjust II
PFS	0.01 (0.00, 0.09) 0.0002	0.00 (0.00, 0.06) < 0.0001
OS	0.01 (0.00, 0.25) 0.0073	0.00 (0.00, 0.20) 0.0056

Adjust I model: adjust for – gender; age.

Adjust II model: adjust for – gender; age, SCr, BUN, ALT, AST, diseases history and other treatment.

### 3.5 Survival curves after EGFR-TKI therapy

**Figure 2:**
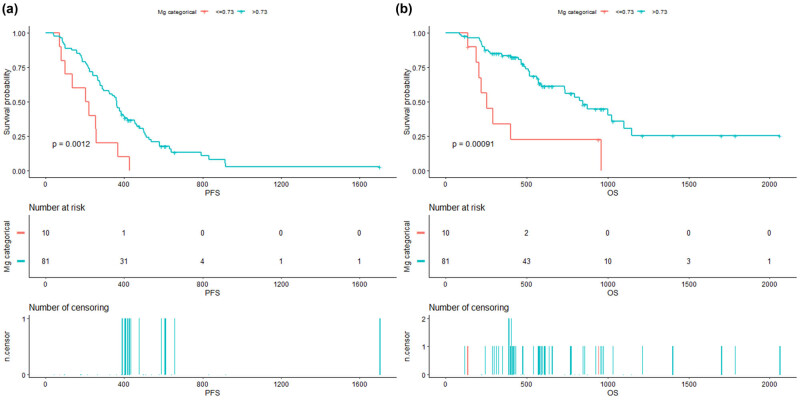
Survival curves after two cycles of EGFR-TKI therapy according to Mg level: (a) PFS before EGFR-TKI therapy; (b) OS before EGFR-TKI therapy.

The surv_cutpoint function within the survival R package was used again to determine the optimal Mg cutoff value for OS, which was 0.73 mmol/L. There were 81 patients (89.01%) in the high Mg group and 10 patients (10.99%) in the low Mg group. The median PFS for patients in the high Mg group was 363 (range: 289–427) days, which was significantly higher than the 204 (range: 136–360) days for patients in the low Mg group. The survival curve revealed that the PFS was significantly prolonged in the high Mg group (*P* = 0.0012). The survival curve for OS produced identical results. The median survival time in the high Mg group (876 days; range: 734–NA days) was higher than that in the low Mg group (346 days; range: 225–876 days). There were significant differences between groups in terms of OS (*P* = 0.00091; Figure 2). The survival R package was used to determine the optimal Mg cutoff levels after therapy. These cutoff levels for serum Mg were lower than the normal range. Therefore, we divided the patients into low (group 1) and high (group 4) groups based on their Mg levels (8–10). The Mg levels were lowest in group 1. The univariate Cox regression analysis was repeated, and related variables were adjusted. The results showed that group 1 was significantly different from the other three groups (Table 6). These outcomes suggest that patients with low Mg levels following TKI therapy have a considerably poorer prognosis compared to other patients.

**Table 6:** Association of serum Mg and prognosis by quartiles of serum Mg

Mg(mmol/L)		PFS		OS	
Groups	Statistics	model I HR (95% CI) *P* value	model II HR (95% CI) *P* value	model I HR (95% CI) *P* value	model II HR (95% CI) *P* value
Group4 (0.94)	25 (27.47%)	1	1	1	1
Group3 (0.86–0.94)	21 (23.08%)	1.57 (0.80, 3.06) 0.1895	1.69 (0.84, 3.40) 0.1414	1.38 (0.54, 3.53) 0.4992	1.36 (0.50, 3.68) 0.5487
Group2 (0.79–0.86)	25 (27.47%)	1.49 (0.77, 2.89) 0.2390	1.87 (0.93, 3.76) 0.0796	1.16 (0.44, 3.04) 0.7630	1.38 (0.51, 3.72) 0.5294
Group1 (−0.79)	20 (21.98%)	2.95 (1.53, 5.67) 0.0012	3.51 (1.75, 7.03) 0.0004	2.84 (1.22, 6.62) 0.0154	3.25 (1.34, 7.91) 0.0094

Adjust I model: adjust for gender and age.

Adjust II model: adjust for gender, age, SCr, BUN, ALT, AST, diseases history, and other treatment.

### 3.6 Interaction test

We further investigated the interactions to elucidate the relationship between Mg levels and the prognosis of EGFR-TKI therapy. Table 7 displays the interactions between Mg levels prior to TKI therapy and other variables. The interaction test results for Mg levels post-EGFR-TKI therapy are shown in Table 8. No variables for PFS interacted significantly with Mg levels either before or after therapy. However, significant variables were identified for OS.

**Table 7:** Interactions between Mg levels before EGFR-TKI treatment and other factors

Variables	PFS						OS					
	*N*	HR	95%CI Low	95%CI High	*P* value	*P* (interaction)	*N*	HR	95%CI Low	95%CI High	*P* value	*P* (interaction)
**Gender**						0.3363						0.0080**
Female	43	0.0013	0.0000	0.4606	0.0265*		43	0.0000	0.0000	0.0008	0.0001***	
Man	48	0.0411	0.0009	1.9443	0.1048		48	0.1667	0.0006	48.4894	0.5360	
**Age**						0.0838						0.0700
<65	56	0.1049	0.0015	7.1149	0.2946		56	0.0235	0.0001	5.6129	0.1794	
≥65	35	0.0003	0.0000	0.0595	0.0028**		35	0.0000	0.0000	0.0093	0.0015**	
**Diseases history**						0.1132						0.0527
No	55	0.0790	0.0014	4.3862	0.2155		55	0.0453	0.0002	8.5738	0.2474	
Yes	36	0.0003	0.0000	0.0739	0.0037**		36	0.0000	0.0000	0.0091	0.0016 **	
**Smoke**						0.9610						0.0173*
No	66	0.0168	0.0005	0.6157	0.0262*		66	0.0621	0.0004	9.4641	0.2785	
Yes	25	0.0139	0.0000	11.7887	0.2139		25	0.0000	0.0000	0.0018	0.0006***	
**Other treatment**						0.4047						0.6347
No	43	0.0019	0.0000	0.5581	0.0307*		43	0.0003	0.0000	0.7868	0.0434*	
Yes	48	0.0361	0.0006	2.0195	0.1057		48	0.0030	0.0000	0.5353	0.0281*	
**BMI**						0.8110						0.5213
Low	45	0.0274	0.0001	5.0882	0.1771		45	0.0007	0.0000	1.4918	0.0631	
High	46	0.0122	0.0002	0.6465	0.0296*		46	0.0130	0.0001	1.6263	0.0779	

*P < 0.05, **P < 0.01, and ***P < 0.001.

**Table 8:** Interactions between Mg levels after TKI treatment and other factors

Variables	PFS						OS					
	N	HR	95% CI Low	95% CI High	*P* value	*P* (interaction)	*N*	HR	95%CI Low	95%CI High	*P* value	*P* (interaction)
**Gender**						0.5738						0.1012
Female	43	0.0019	0.0000	0.1430	0.0045**		43	0.0006	0.0000	0.1345	0.0074**	
Man	48	0.0092	0.0003	0.3022	0.0085**		48	0.3237	0.0016	64.8369	0.6766	
**Age**						0.2887						0.1043
<65	56	0.0160	0.0005	0.5472	0.0218*		56	0.1183	0.0008	17.5948	0.4030	
≥65	35	0.0010	0.0000	0.0478	0.0005***		35	0.0004	0.0000	0.0469	0.0014**	
**Diseases history**						0.2900						0.0672
No	55	0.0160	0.0005	0.5584	0.0225*		55	0.1503	0.0013	17.2631	0.4336	
Yes	36	0.0009	0.0000	0.0487	0.0006***		36	0.0001	0.0000	0.0449	0.0026**	36
**Smoke**						0.6774						0.0183*
No	66	0.0033	0.0001	0.0737	0.0003***		66	0.1332	0.0019	9.3364	0.3525	
Yes	25	0.0133	0.0000	4.6354	0.1481		25	0.0000	0.0000	0.0084	0.0020**	
**Other treatment**						0.4456						0.7159
No	43	0.0013	0.0000	0.0739	0.0012**		43	0.0138	0.0001	2.5967	0.1089	
Yes	48	0.0106	0.0003	0.3967	0.0139*		48	0.0035	0.0000	0.6815	0.0355*	
**BMI**						0.6905						0.0990
Low	45	0.0029	0.0001	0.1464	0.0035 **		45	0.0002	0.0000	0.0766	0.0046**	
High	46	0.0086	0.0002	0.3369	0.0110*		46	0.1261	0.0012	13.1979	0.3828	

^*^ *P* < 0.05, **P < 0.01, and ***P < 0.001.

For pre-treatment Mg levels, the interaction test revealed that, compared to non-smokers, the Mg levels of smokers were more strongly associated with OS as a protective factor (HR < 0.0001 vs HR = 0.0621; *P* = 0.0173). Similarly, Mg levels were more strongly correlated with the outcomes of oral EGFR-TKI therapy in women than in men (HR < 0.0001 vs HR = 0.1667; *P* = 0.008).

Post-therapy, Mg levels demonstrated a specific predictive effect for OS only in smokers (HR < 0.0001 vs HR = 0.1332; *P* = 0.0183), but not for gender. Although there was no statistically significant difference in OS between females and males (HR = 0.0006 vs HR = 0.3237; *P* = 0.1012), women exhibited unique characteristics.

## 4 Discussion

The majority of patients eventually develop resistance to EGFR-TKI drugs, leading to disease progression. Consequently, most patients require regular evaluations using CT and nuclear imaging. However, the limitations of imaging techniques in capturing cellular metabolism hinder the timely detection of disease progression and the modification of treatment plans. Therefore, exploring the relationship between tumor-specific or other relevant blood markers and prognosis following EGFR-TKI therapy is rational [12,13].

Compared to other studies, in our study, we examined the data after two treatment cycles. Based on the results, we propose that patients with extremely low Mg levels following TKI therapy have a significantly poorer prognosis compared to other patients. Furthermore, the interaction test revealed that reduced Mg levels are more likely to be a risk factor in patients with a history of smoking. This observation aligns with previous research, which identified hypomagnesemia as a prognostic indicator of unfavorable outcomes in chemoradiotherapy for head and neck cancer [8]. Similarly, clinical studies suggest that depleted Mg levels constitute a risk factor for hepatocellular carcinoma among patients with nonalcoholic fatty liver disease [14].

Mg, an abundant bivalent cation in the human body, is primarily stored in skeletal muscle, soft tissue, and bone. Hormones or drugs rarely directly affect Mg levels, which are primarily regulated by intestinal absorption and renal excretion [15]. Mg is absorbed through two channel proteins: transient receptor potential melastatin (TRPM) channel types 6 and 7. TRPM7 is widespread, whereas TRPM6 is the primary absorption channel for Mg and is found primarily in the kidneys, distal small intestine, and colon [16]. The epidermal growth factor (EGF) and its receptor (EGFR) regulate the activity of the Mg channel TRPM6. Abnormal activation of EGF causes TRPM6 in the cytoplasm of renal cells to move toward the cell membrane, resulting in increased Mg reabsorption [17,18].

Consequently, EFGR inhibitors could theoretically affect magnesium absorption and induce hypomagnesemia. Consistent with this theory, several studies have reported that the treatment of tumors with EGFR monoclonal antibodies induces hypomagnesemia. Patients treated with cetuximab, alone or in combination with other therapies, have been reported to experience hypomagnesemia [19,20,21]. Anti-EGFR antibodies inhibit TRPM6 activity, reduce Mg influx into cells, and inhibit renal Mg reabsorption. Mutations in the TRPM6 gene, which encodes the epithelial Mg channel, result in secondary hypocalcemia; changes in renal function also affect Mg levels [22,23]. Therefore, after adjusting for liver and kidney function, K, Ca, and other electrolytes in the multiple regression analysis, the outcomes of our study remained relatively unchanged. This further demonstrated the predictive nature of Mg in EGFR-TKI treatment.

So far, no significant clinical trials have demonstrated a clear correlation between EGFR-TKI (gefitinib, erlotinib, or lapatinib) and hypomagnesemia [24,25]. Unlike anti-EGFR monoclonal antibodies, the precise effect of EGFR-TKI on hypomagnesemia remains unclear. Dimke et al. demonstrated that erlotinib can alter EGF-stimulated TRPM6 channel activity at the cellular level. However, animal studies indicate that normal therapeutic doses of erlotinib have no significant effect on serum Mg levels in humans [26].

Mg is a crucial element required to form the active structures of protein kinases, including receptor tyrosine kinases [27]. Cellular studies indicate that high Mg levels can increase tyrosine kinase activity, as both enzyme activity and phosphate transfer require Mg molecules. The Mg-adenosine triphosphate complex binds protein substrates and catalyzes energy for signaling pathway transmission [28,29,30]. Thus, it can be inferred that Mg levels promote EGFR-related pathways. Thus, it can be inferred that Mg levels promote EGFR-related pathways. We hypothesize that patients with elevated serum Mg levels who are treated with EGFR-TKI may have longer PFS and OS based on the following: EGFR mutations lead to increased Mg absorption; that is, elevated Mg levels indicate that EGFR mutations account for a higher proportion of tumor development, thereby increasing the sensitivity of EGFR-TKI treatment. Further research is required to confirm these hypotheses.

Interaction tests demonstrated that gender and smoking factors interacted with Mg levels, while smoking alone interacted with Mg levels both before and after therapy. In a large clinical study of esophageal cancer, the interaction between Mg intake and smoking was statistically significant [31]. However, this study showed a strong positive correlation between Mg and the incidence of esophageal adenocarcinoma among non-smokers than among smokers. In our study, smokers also represented a special group. In patients with prognostic risk factors, such as those over 65 years of age, those with a history of hypertension and other diseases, and those with a low BMI, Mg tended to be a protective factor for OS, although this was not statistically significant.

Chemotherapy utilizing platinum-based agents can impair renal function and result in hypomagnesemia, while anti-EGFR monoclonal antibody therapy can affect Mg absorption. Furthermore, several studies have demonstrated that magnesium supplementation prior to or during treatment enhances patient outcomes [32,33,34]. A study by Minzi et al. revealed that supplementing electrolytes can reduce the risk of nephrotoxicity caused by chemotherapy with cisplatin [32]. Therefore, in combination with our study results, we hypothesize that exogenous electrolyte supplementation during EGFR-TKI therapy may affect therapeutic efficacy, particularly in patients with a history of smoking and other risk factors. However, no studies have reported this previously; therefore, additional clinical trials are required to verify these results.

Our research had several limitations. First, it was a retrospective study with a limited sample size; therefore, bias was inevitable. Second, we did not investigate the effect of electrolyte supplementation on prognosis during therapy. Third, additional follow-up is required to determine the impact of a steady increase in Mg levels on treatment outcomes.

## 5 Conclusion

Our findings indicate that maintaining a high Mg level is a significant predictor of PFS and OS in patients with NSCLC undergoing EGFR-TKI therapy. Smokers may represent a unique population demonstrating a significant relationship between OS and Mg levels. These results provide new insight into the underlying mechanisms of EGFR-TKI therapy associated with electrolyte balance.
